# The Formation of Urea

**Published:** 1871-09

**Authors:** 


### The Formation of Urea.

The methods thus far adopted (by Prevost and Dumas, Oppler, Perls, Zaltsky, and Grehaut), to prove or disprove the part taken by the kidneys in the formation of urea, are based principally upon the examination of the blood of the renal vessels passing to or from the kidneys, or that of the general system, with a view to its changeable proportion of urea with retained or extirpated kidneys, or with extirpation of the kidneys with litigation of the ureters.—The uncertainty of all the modes of fixing the amount of urea in the blood induced Dr. Rosenstein to adopt a new method for the purpose. He compared the amount of urea in the urine of dogs, with the same food, after the retention of both kidneys, and the extirpation of one. The result was, that where one kidney only was left, the same amount, and generally even more urea was secreted, than where both were retained. As long as this result was obtained only some time after the extirpation of the single kidney, it was convincing, because in the mean while the remaining kidney was decidedly enlarged, and an augmented compensatory function through an increase of tissue or of secretory elements might be assumed. Meanwhile Dr. Rosenstein obtained the same result in one animal which before the operation had been put on full diet, and whose desire for food was by no means diminished by the operation. In this case the elimination of the urea, in both the days following the operation, was the same as before, when both kidneys were present. Since in these two days no noticeable increase of size took place in the remaining kidney, it certainly follows that the kidneys do not take part in the formation of urea.—*Centralblatt Medizinischer Wissenschaften,* No. 2371.—*N. Y. Med. Journal.*

